# Outcrossing Complicates Mutation Purging by Trapping Single Nucleotide Polymorphisms in Structural Variant Mutations

**DOI:** 10.1101/2025.04.16.649179

**Published:** 2025-08-13

**Authors:** R. Kapila, S. Saber, R.K. Verma, G. Blanco, V.K. Eggers, J.L. Fierst

**Affiliations:** 1 Department of Biological Sciences, Florida International University, 11200 8th Street, Miami, 33199, FL, USA; 2 Biomolecular Sciences Institute, Florida International University, 11200 8th Street, Miami, 33199, FL, USA

**Keywords:** Mutations, Outcrossing, Structural variations, Genome, *Caenorhabditis elegans*

## Abstract

Classical mutational theories centered on single nucleotide polymorphisms suggest that outcrossing enhances the purging of deleterious mutations by promoting recombination. However, larger structural variants, such as insertions, deletions, and inversions, can suppress recombination and create linkage blocks. In this study, using experimental evolution in *C. elegans* lines and whole-genome sequencing, we show that outcrossing populations struggle to purge structural mutations and instead retain mutations small and large as single nucleotide polymorphisms become trapped within larger structural variants. Supporting this, our population genetic simulations showed that structural variants accumulate more readily under higher outcrossing rates. These findings contradict classical population genetic expectations, showing that outcrossing can hinder the removal of structural mutations and complicate mutation purging by trapping small variants within larger mutations. Together, our results challenge the prevailing view that sex uniformly promotes genomic resilience and reveals a previously underappreciated constraint on mutation purging.

## Introduction

The accumulation of deleterious mutations can have severe consequences for populations, leading to long-term fitness decline or, in extreme cases, extinction (Lynch et al., 1993). The ability to effectively eliminate or purge harmful mutations is critical for reducing genetic load and ensuring long-term evolutionary fitness (Lynch et al., 1999). Based on their size and scale, mutations are broadly categorized into two types: single nucleotide polymorphisms (SNPs) and structural variants (SVs). SNPs are small-scale changes involving a single base pair, often resulting in localized effects on amino acids or regulatory elements (Robert & Pelletier, 2018). In contrast, SVs are large-scale genomic changes including deletions, duplications, inversions, and translocations. These can impact larger portions of the genome, frequently altering multiple genes or regulatory networks (Feuk et al., 2006).

While mutation purging operates on both SNPs and SVs, the process may be more straightforward for SNPs due to their smaller scale, localized effects, and simpler genomic dynamics (Collins et al., 2020). In contrast, purging SVs may be more complex due to their larger genomic footprint and ability to suppress recombination (Mahmoud et al., 2019). Evolutionary theory has traditionally viewed the spectrum of genomic changes under the broad category of “mutation”. Much of the research on mutation purging has disproportionately focused on SNPs as they are more easily and accurately detected (Ho et al., 2020). However, the advent of large-scale genome sequencing has revealed the ubiquity of SVs. Despite the foundational role of mutation purging in evolutionary theory (García-Dorado, 2012; Glémin, 2003; Whitlock & Agrawal, 2009),our understanding of how it operates on SVs remains unexplored. Addressing this is essential to refining theoretical models, validating predictions with empirical evidence and understanding how mutation size and severity interact with established evolutionary predictions.

Mating systems influence mutation purging by affecting genetic diversity, recombination, and the expression patterns of deleterious mutations (Byers & Waller, 1999; Szövényi et al., 2014). Current consensus in the field is that outcrossing populations, through recombination, create novel genetic combinations which facilitates the purging of deleterious variation (MacPherson et al., 2021). However, outcrossing can also potentially dilute the effects of deleterious mutations and slow their purging (Sianta et al., 2023). Reduced recombination and genetic diversity in populations that reproduce through self-fertility may lead to the accumulation of mildly deleterious mutations through genetic drift (Hartfield, 2016). Selfing however, can also increase homozygosity, exposing harmful recessive mutations to selection and enabling more effective purging (Hartfield, 2016). These processes may differ for mutations of different physical sizes. SNPs, with largely localized effects, may be more directly exposed to selection. Purging deleterious SVs may be complicated by their larger size and diverse impacts, both of which can vary significantly with mating system (Hoffmann & Rieseberg, 2008; Kirkpatrick & Barton, 2006). Outcrossing can facilitate purging through recombination but this process could be physically disrupted by SVs, which are capable of suppressing recombination and creating linkage blocks. The complex interplay between mating system, recombination suppression by SVs, and mutation purging is not well understood.

In this study we use experimental evolution to address two key questions: First, how do levels of outcrossing influence the genome’s ability to purge deleterious mutations? Second, how does this ability vary with mutation size and genomic location? To investigate these questions, we experimentally evaluated how three strains of *Caenorhabditis elegans*, which differ in natural male frequency (N2, AB1, and CB4856) respond to mutagenesis and recovery. As an androdiecious species *C. elegans* populations consist of both males and hermaphrodites, allowing for reproduction through either self-fertilization or outcrossing with males. While self-fertilization predominates in *C. elegans*, the frequency of males and outcrossing varies drastically across different strains (Gaertner & Phillips, 2010) making *C. elegans* a prime system to test these ideas.

We subjected *C. elegans* strains to two contrasting mutagens, EMS to induce single nucleotide mutations and formaldehyde to introduce complex lesions in the genome (Kutscher & Shaham, 2014). We mutagenized worms for five consecutive generations and allowed them to recover from induced mutations for three generations. We then tracked the population’s relative fitness, changes in male frequency, and outcrossing rates to investigate their ability to cope with mutational input and purge deleterious mutations. We employed both long-read and short-read sequencing to quantify the size, location, and types of mutations that persist after recovery.

## Results

### Experimental test of the outcrossing prediction

To test the impact of outcrossing on mutations we subjected three strains of *C. elegans* (N2 (Bristol), AB1, and CB4856 (Hawaiian)), obtained from the *Caenorhabditis* Genetics Center, University of Minnesota) to five generations of mutagenesis followed by three generations of recovery in large, high-density populations. AB1 and CB4856 are wild isolates, whereas N2 is a long-term laboratory adapted strain, allowing us to test the impact of mutagenesis on diverse genetic backgrounds (Brenner 1974; Sterken et al. 2015). Each strain was maintained in two distinct lineages: one mutagenized with EMS and the other with formaldehyde, with four replicate populations per lineage.

### Male frequency was differentially affected by mutagenesis

**Figure 1.**
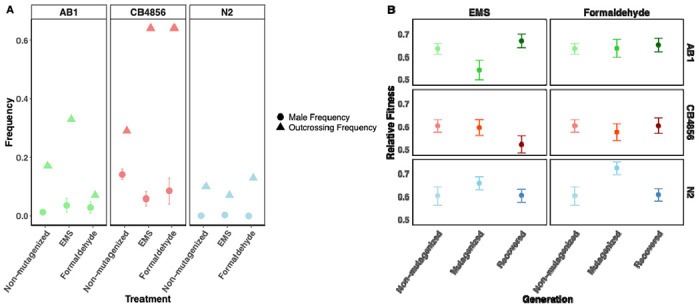
Male frequency, outcrossing behaviour, and relative fitness reveal strain-specific responses to mutagenic stress in *C. elegans*. (A) Mean male frequency across strains, represented by solid circles, before mutagenesis (non-mutagenized) and following EMS or formaldehyde exposure. CB4856 exhibited the highest male frequency across all treatments, followed by AB1 and N2. Outcrossing frequency, shown as solid triangles, was consistently higher in CB4856 across treatments, suggesting strain-specific differences in mating behaviour. (B) Relative fitness across strains and generations, comparing non-mutagenized (lightest shade), mutagenized (medium shade), and recovered (darkest shade) populations. AB1 (green) demonstrated the highest post-mutagenesis resilience, while CB4856 (red) showed the lowest recovery in fitness.

Non-mutagenized CB4856 populations had the highest male frequencies (Figure 1A), significantly greater than either AB1 (*p* < 0.0001) or N2 (*p* < 0.01). Mutagenesis had a strain-specific effect on male frequency. In CB4856 populations male frequency was reduced after mutagenesis with EMS (*p* = 0.03; Figure 1A) and formaldehyde, although this comparison was not statistically significant (*p* = 0.11). In N2 populations, male frequency did not change in response to either mutagen (*p* = 1.00 for both). Similarly, AB1 male frequencies did not differ across populations or treatments (all *p*-values were > 0.05; Supplementary Material Table 1; Figure 1A).

### Outcrossing frequencies varied between C. elegans strains

We found that CB4856 populations had the highest outcrossing frequencies under all conditions, with peaks following mutagenesis (Figure 1A). In contrast, N2 populations displayed the lowest outcrossing frequencies. AB1 populations were intermediate to these with notably higher values in EMS mutagenized populations compared with both non-mutagenized strains and the AB1 formaldehyde mutagenized population.

### C. elegans populations quickly recovered fitness

Three generations of recovery from mutagenesis were sufficient to restore fitness to premutagenesis levels across all strains (Figure 1B). For CB4856 relative fitness was not affected by formaldehyde (p=1.00) or EMS (p=0.18). Similarly, for AB1 pre-mutagenesis and post-recovery fitness did not change with treatment by either formaldehyde (p=0.94) or EMS (p=0.76). The initial response to mutagenesis was different for N2 populations which showed an increase in relative fitness immediately after exposure to formaldehyde (p<0.03) but not EMS (p<0.46). However, N2 relative fitness decreased to pre-mutagenesis levels after recovery (p=1.00).

### Genomic analyses showed numerous SVs and SNPs were retained after recovery

Despite the rapid fitness recovery, sequence analyses showed that populations harbored numerous *de novo* mutations. To comprehensively identify SVs and SNPs we sequenced whole genome DNA libraries with both Pacific Biosystems long-read and Illumina short-read DNA sequencing. Short-read data was used to characterize SNPs and both short- and long-read data were used to generate a high-confidence SV dataset.

**Figure 2:**
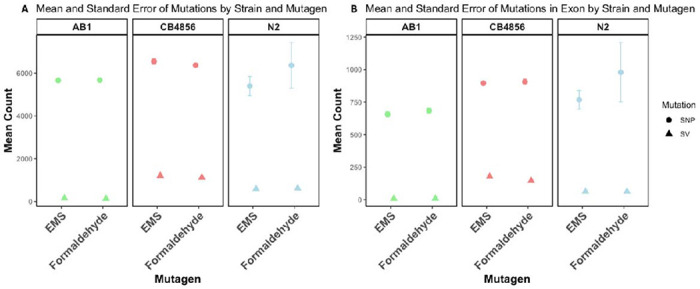
Comparison of de novo SNPs and SVs mutations and their localization in exons across strains (A) Mean counts of de novo SNPs (Circles) and SVs (Triangles) induced by EMS and Formaldehyde across AB1 (Green), CB4856 (Red), and N2 (Blue). De novo SNPs are significantly more abundant than de novo SVs under both mutagen treatments. CB4856 has the maximum number of de novo SNPs and de novo SVs. (B) Mean number of de novo SNPs and de novo SVs localized in exons for the same strains and mutagen treatments, with de novo SNPs again being more prevalent than de novo SVs and CB4856 having more SVs in exons than the other two strains and more SNPs in exons than AB1. Compared to N2, CB4856 had more SNPs in exon than N2 when treated with EMS and comparable SNPs in exon when treated with Formaldehyde.

We found that CB4856 exhibited significantly higher susceptibility to mutagenesis compared to AB1. When mutagenized with formaldehyde, CB4856 populations showed a 1.12-fold increase in *de novo* SNPs compared to AB1 (t=5.55, *p*=0.0014) and comparable SNP counts to N2 (t < 0.01, *p*=0.996). In contrast, with EMS as the mutagen, CB4856 showed a significantly higher *de novo* SNP count when compared with AB1 (t = 6.79, p < 0.01) and a marginally higher *de novo* SNP count when compared with N2 populations (t = 2.45, p = 0.05; Figure 2A).

Due to marked heteroscedasticity in SV counts we performed Mann–Whitney U tests to compare *de novo* SV counts among strains. Under both EMS and formaldehyde treatments, CB4856 populations had significantly higher *de novo* SV counts (Figure 2A) when compared with AB1 (W=16, *p* = 0.03 for both treatments) and N2 populations (W=16, *p* = 0.03 for both treatments).

### CB4856 harbored high numbers of SNPs and SVs in exons

**Figure 3:**
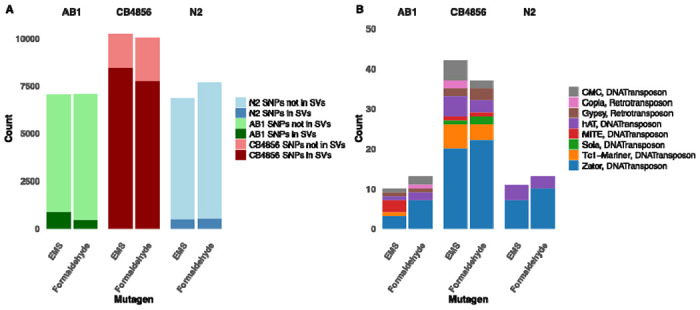
Mutational and TE landscape across strains and mutagens **(A)**: Number of SNPs, SVs and SNPs in SVs for AB1, CB4856 and N2; **(B)**: Classification and count of active TE families contributing to transposition events across strain-treatment combinations. DNA transposons such as Zator and Tc1-Mariner were among the most active elements, with CB4856 showing the broadest TE activity profile.

CB4856 populations had the highest number of mutations in coding regions (Figure 2B) and the highest number of SNPs trapped within SVs (Figure 3A). When formaldehyde was used as the mutagen, CB4856 had 2.20 times more exonic SNPs than AB1 (W=0, *p*= 0.03) and 1.12 times more than N2 (W=12, *p*=0.34). Similarly, when mutagenized with EMS, CB4856 had 2.26 times more exonic SNPs compared to AB1 (W=0, *p*=0.03) and 1.38 times more exonic SNPs compared to N2 (W=16, *p*=0.03) (Figure 2B).

Exons were similarly affected by SVs. AB1 populations showed the least number of exons containing SVs, whereas CB4856 populations had the highest number. AB1 formaldehyde populations had 16.25-fold fewer exonic SVs than CB4856 (W = 0, p = 0.02) and 6.92-fold fewer than N2 (W = 16, p = 0.03). CB4856 EMS populations had 22.31-fold more exonic SVs compared to AB1 (W = 0, p = 0.02), and N2 had 7.81-fold more when compared with AB1 (W = 16, p = 0.03) (Figure 2B).

We found that CB4856 had the highest number of SNPs trapped within SVs (77.13% for formaldehyde and 82.46% for EMS treatment; Figure 3A). In contrast, SVs in AB1 populations contained 6.36% (formaldehyde) and 12.01% (EMS) of *de novo* SNPs. N2 populations had similarly low numbers of SNPs contained in SVs (EMS 7.12%; formaldehyde 6.80%).

### Active transposable elements were responsible for a small portion of SV mutations

Both mutagenesis and outcrossing can activate TEs. To determine if TE activity was responsible for the SVs in our mutagenized populations we used TransposonUltimate (Riehl et al., 2022) to annotate repeats and TEs. We compared populations pre- and post- mutagenesis to both analyze *de novo* TE-associated SV mutations and identify potentially active TEs (Riehl et al., 2022).

We found that *de novo* TE-associated SVs constituted a small proportion (<15% in all populations) of the total *de novo* SVs. The parental AB1 population had the highest genomic repeat content (25.71%) but the fewest TE-associated SVs (Figure 3B). In contrast, CB4856 had the lowest genomic repeat content (21.68%) and the most TE-associated SVs. The N2 parental population had 23.19% genomic repeat content. Some TEs, including Copia, Gypsy, and Sola in CB4856, were strain-specific or uniquely active in one population. Others like the DNA transposon Zator (Figure 3B), were consistently active in all populations and across both mutagens. Zator was responsible for over half of the TE-associated SVs in CB4856, N2 and formaldehyde-mutagenized AB1 populations.

### Mutagenesis induced large SV mutations

**Figure 4:**
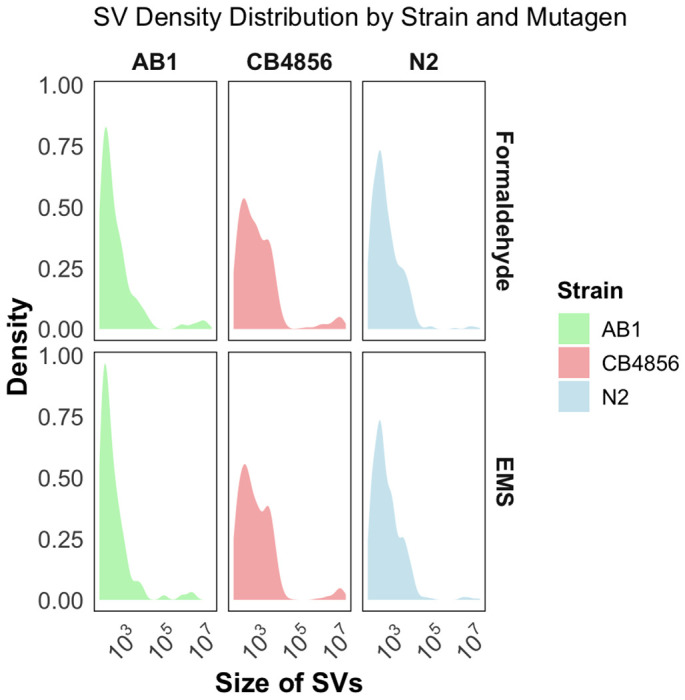
Size distribution of structural variants (SVs) across *C. elegans* strains and mutagens: Density distribution of SVs sizes for all the three strains for both the mutagens. x- axis Size of SVs, Y-axis: density of SVs.

We found that while most SVs were small (<10^4^ bp), both formaldehyde and EMS induced numerous large mutations and affected substantial portions of the genome (Figure 4). In the N2 formaldehyde-treated population, insertion mutations created 471,238 nucleotides of new genetic material, approximately 0.48% of the 100.81Mb *C. elegans* genome (Supplementary Material Table 3). In comparison, 74,044 nucleotides (0.06%) were deleted, 44,038 nucleotides (0.04%) were duplicated, and 1,289,368 nucleotides (1.29%) were affected by inversions. Mutations in the EMS-mutagenized N2 populations were of similar scale.

AB1 populations showed the smallest number of nucleotides affected by SV mutations. In formaldehyde-treated AB1, 35,468 nucleotides were inserted and 46,363 deleted. The notable exception was inversion mutations, which spanned 2,539,375 nucleotides in formaldehyde-treated and 4,181,603 nucleotides in EMS-treated populations. CB4856 populations harbored substantially more DNA affected by structural variation. Roughly 1 Mb of new DNA was inserted under both treatments (Supplementary Material Table 3). Compared to AB1, CB4856 had 14.11-fold more insertions under formaldehyde and 15.18-fold more under EMS. Relative to N2, it had 1.50-fold and 1.66-fold more insertions under formaldehyde and EMS, respectively. CB4856 also had large amounts of duplicated DNA: 1.2 Mb under formaldehyde and 2.1 Mb under EMS. Strikingly, inversion mutations accounted for 49.7 Mb—nearly half the genome—in formaldehyde-treated CB4856, including large 13 Mb and 8 Mb inversions, with additional megabase-scale and smaller rearrangements. In EMS-treated CB4856, inversions spanned 32.8 Mb—almost a third of the genome (Figure 4).

### SVs and transposon activity varied across chromosomes

**Figure 5:**
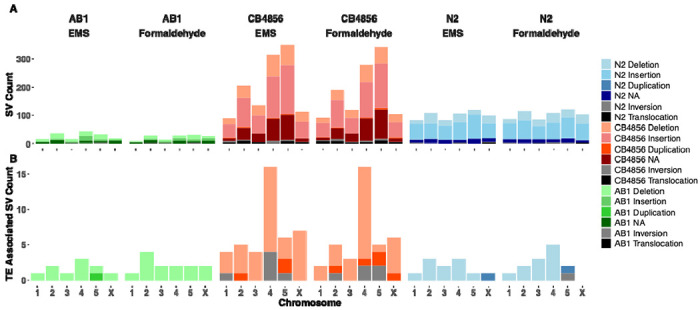
Chromosomal distribution of SVs and TE-associated SVs across strains and mutagens. (A) Stacked bar plots showing the total count and types of SVs including Deletions, Insertions, Duplications, Inversions, Translocations, and Unclassified (NA) across chromosomes 1–5 and X, for each strain (AB1, CB4856, N2) and mutagen (EMS and formaldehyde). (B) TE associated SVs were primarily Deletions.

The distribution of SVs varied across chromosomes, regardless of strain or mutagen (Figure 5A). SV mutations occurred in higher numbers on Chromosome IV and V and lower numbers on Chromosome X. TE activity did not explain these patterns as, for example, TEs were most active on Chromosome IV in CB4856 but Chromosome V harbored the highest number of SVs (Figure 5B). TE-associated duplications and inversions were less frequent than deletions across all populations. Additionally, we found shared transposition events between the two mutagens. The highest overlap between formaldehyde and EMS TE-associated mutations was observed in N2 where 33.3% of the TE-associated mutations were shared. In CB4856 17.9% of the TE-associated mutations were shared between EMS and formaldehyde treated populations while in AB1 it was just 4.5%. These findings indicate that while TEs contribute to structural variation, they do not account for the full spectrum of observed SVs.

### Simulation support of experimental results

Although there is a body of work modelling the influence of outcrossing on mutation in populations these models typically assume that mutations are occurring at the level of an individual SNP or broader, abstract ‘allele’ level (Kondrashov, 1982; Charlesworth, 1990a). To explore whether our experimental results could emerge under different evolutionary scenarios, we developed a population model describing evolution of SVs in the genome. We implemented this model in stochastic simulations using the SLiM 4.3 software (Haller & Messer, 2023) and measured the impact of outcrossing rates on SVs in the population.

**Figure 6.**
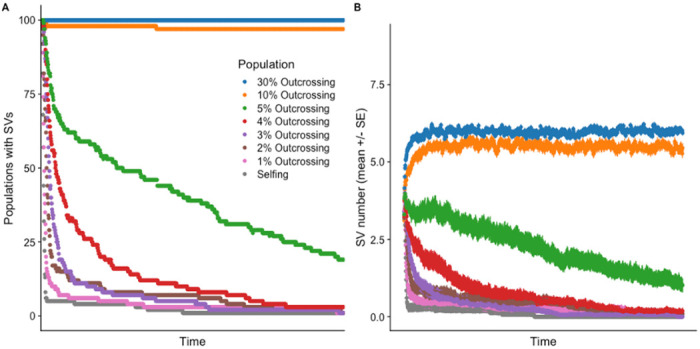
A) Simulated populations evolving with less than 10% of individuals reproducing through outcrossing do not retain SVs. B) The mean number of structural variant mutations is lower in populations with lower quantitative levels of reproduction through outcrossing. Here *K*=1000, =0.001, =0.0006, =0.01, =0.001 and recombination rate =0.0001.

Consistent with our experimental observations, the simulations showed that outcrossing populations retained SVs, particularly when outcrossing rates consistently exceeded 30%. In contrast, fully selfing populations rapidly purged SVs. SVs were retained in outcrossing populations when each mutation carried a small deleterious effect on fitness, drawn from a gamma distribution (Supplementary Figure 1; Eyre-Walker & Keightley, 2007; Keightley & Eyre-Walker, 2007; Racimo & Schraiber, 2014). The fitness landscape had a negative curvature or synergistic epistasis (Supplementary Figure 2). Under these conditions, SVs persisted in simulated populations. These results suggest that outcrossing can, under specific conditions, promote the persistence of structural mutations (Figure 6).

## Discussion

In this study we investigated how quantitative levels of outcrossing influence the genome’s ability to purge deleterious mutations and how this ability varies with mutation size and genomic location. Leveraging the unique mating system of *C. elegans*, we combined experimental evolution with genomic analyses to assess how outcrossing influences the purging of mutations of different sizes and locations. We found that CB4856, characterized by highest male frequency, outcrossing rate and elevated TE activity, regained fitness after mutagenesis and recovery. Despite this, CB4856 populations retained large numbers of *de novo* SNPs and SVs. Importantly, we found many SNPs were trapped within SVs and overlapped with exonic regions, making it difficult to purge these variants through recombination and outcrossing.

*C. elegans* is one of the most tractable model systems and has emerged as a powerful model organism for unraveling the intricate dynamics of mutation accumulation. Traditionally, researchers have employed two primary approaches to investigate this phenomenon. The first approach involves manipulating DNA repair pathways, creating “mutation accumulation” lines with compromised repair mechanisms (Estes et al., 2024). This allows for controlled observation of how increased mutation rates impact population fitness and adaptation over generations. The second approach takes a more observational stance, tracking the accumulation and consequences of naturally occurring mutations over extended periods (Konrad et al., 2017). Both approaches have yielded valuable insights into the remarkable ability of *C. elegans* populations to tolerate and potentially recover from mutational stress. However, despite these advancements, a crucial aspect remains underexplored: how mating system variation and reproductive strategies interact with mutation size and the resulting impacts on genomic variation.

We explored these factors in our population simulations and found that under specific assumptions increased levels of outcrossing would retain SVs. Importantly, the phenotypic effect size of the segregating mutations was very small. In a fully outcrossing simulated population, there was an average of 10 mutations per individual after 100,000 generations, each with an average phenotypic effect size of 0.0035. Larger mutation effect sizes were rapidly purged from outcrossing populations. However, deleterious mutations with small effects on the phenotype and fitness were stably maintained in the simulated outcrossing populations for long periods.

The mechanisms by which genomes cope with mutational threats are multifaceted and depend heavily on the type of lesion incurred. Point mutations like SNPs predominantly affect gene expression and protein structure and are typically repaired through efficient pathways like base excision repair (BER) or nucleotide excision repair (NER). In contrast, complex lesions like SVs (structural variants) pose a more significant challenge to genome stability, often leading to chromosomal aberrations (Ciccia & Elledge, 2010). Repairing these intricate injuries often relies on more intricate mechanisms like homologous recombination (HR) and non-homologous end joining (NHEJ) (Limpose et al., 2017). In our study we used two types of mutagens that are known to affect the genome in different ways. EMS is known to cause more point mutations and formaldehyde is known to cause complex lesions. We expected that EMS should be creating more SNPs and formaldehyde should be causing more structural variations in the genome. However, we found that long term mutagenic abuse (5 consecutive generations in our case) with either of the mutagen had similar mutational effects on the *C. elegans* genome and resulted in SNPs and SVs ranging from 10s of nucleotides to multiple megabases in length (Supplementary Material Table 3).

The potential impact of male frequency and outcrossing on mutational burden remains highly debated. Studies in dioecious species suggest that intense male competition for mates drives stronger selection against deleterious mutations, potentially benefiting populations facing genomic stress (Whitlock & Agrawal, 2009). However, bottlenecks arising from such strong selection can ironically counteract this advantage by allowing the accumulation of detrimental mutations. Additionally, the relevance of these findings to androdiecious species like *C. elegans* is unclear. Due to their self-fertilizing nature, *C. elegans* males might not experience the same level of selective pressure as those in dioecious species (Artieri et al., 2008). Consequently, increased male frequency in *C. elegans* might not directly translate to mutational resilience. Nevertheless, higher male proportions could offer another advantage: increased outcrossing, which can introduce new genetic variation. This influx of novel alleles could enhance the population’s adaptability and resilience in the face of unpredictable challenges like ongoing mutagenic stress. Moreover, mutation purging processes differ significantly between self-fertilizing and outcrossing mating systems, particularly for SNPs and SVs. In self-fertilizing populations, increased homozygosity facilitates rapid purging of highly deleterious mutations from the genome. However, reduced genetic variation weakens selection against slightly deleterious mutations, potentially leading to the accumulation of slightly deleterious SNPs and SVs. In contrast, outcrossing populations can maintain both highly deleterious and slightly deleterious mutations if they are recessive.

Our results suggest that there are strain- and mutagen-specific effects, with CB4856 showing the highest male frequency among the three strains. However, it exhibited the worst relative fitness after three generations of recovery from mutagenic treatment and carried the highest mutational load for both mutagens. AB1, in contrast, had second largest male frequency but was least affected by mutagenic abuse and had the minimum mutagenic load in the genome when compared with the other two strains. N2 had the lowest male frequency but the highest relative fitness during mutagenesis; however, its fitness declined during recovery, suggesting that the elevated fitness under mutagenic stress may have come with costs that became apparent in a stable environment.

Strain-specific differences in coping with mutagenic stress may reflect distinct genomic architectures. For example, AB1 had few SNPs within SVs and the lowest exonic overlap of all strains, potentially minimizing disruption to coding regions. This pattern could act as a genomic “buffer,” reducing the functional impact of mutations and contributing to resilience after mutagenic stress.

CB4856 had the highest number of de novo SNPs and SVs. A high proportion of SNPs were trapped within SVs and a significant number of these mutations intersected with exonic regions, potentially disrupting essential genes. This genomic configuration likely explains CB4856’s reduced resilience under mutagenic stress, as the interplay between structural and single nucleotide variants can amplify the deleterious effects of mutations, complicating the purging process. The broad and evenly distributed mutational landscape especially for SV ranging from 1,000 base pairs to 10^7^ base pairs (Fig. 4) may favor greater genomic plasticity, improving a species generation of genetic variation and potential adaptation in uncertain environments. However, CB4856 experienced the most pronounced decline in fitness following mutagenesis. The delicate balance between evolutionary plasticity and mutational resilience may tip toward instability under the stress of chemical mutagenesis.

N2 populations showed relatively stable relative fitness responses without significant increases in male frequencies or outcrossing frequencies and relatively low genomic responses to mutagenesis. This suggests that N2 relied heavily on DNA repair mechanisms, rather than increased genetic variation through outcrossing, to mitigate the negative effects of induced mutations. The combination of self-fertilization and the retention of small SVs may minimize genomic disruptions, ensuring genomic integrity over generations. This may balance minimizing risk through self-fertilization and small SV accumulation, while still allowing for some level of genomic flexibility. This type of response may allow an organism to persist in environments where maintaining genomic integrity is essential for survival. Interestingly, the relative fitness of N2 with both the mutagens increased which came back to the pre-mutagenizes levels after recovery phase. This suggest that the initial increase in fitness might come with a long-term cost, potentially limiting its adaptive potential over time.

Transposable elements (TEs) can play a critical role in shaping genomic resilience by introducing genetic variation and driving structural changes in response to stress (Negi et al., 2016). Increased outcrossing has been associated with elevated TE activity, likely due to the enhanced recombination rates and reduced efficiency of silencing mechanisms in outcrossing populations (Dolgin & Charlesworth, 2006; Wright & Schoen, 1999). Our results highlight significant strain-specific differences in TE activity post-mutagenesis. TEs like Tc1, known for their cut-and-paste transposition mechanism and historical activity in *C. elegans* (Plasterk, R. H. A, 1996), were not the dominant contributors to SVs in our findings. In fact, in N2 and AB1 under formaldehyde treatment, Tc1 was entirely absent, yet these strains still retained substantial structural variation, indicating that SV retention cannot be attributed solely to Tc1 or any other single transposable element’s activity. CB4856, with the highest outcrossing frequency, exhibited the highest transposition rates, reflecting its propensity for increased genomic instability under mutagenic stress. Interestingly, although AB1 had the highest number of annotated transposons, its transposition rates were lower, suggesting fewer active TEs. Elevated TE activity in CB4856, coupled with unique transposons such as Copia, Gypsy, and Sola, may indicate a potential for adaptability through increased genetic variation and a capacity to respond to environmental challenges. Interestingly, N2 exhibited the highest overlap in TE events between mutagens (33.3%), suggesting common TE activations under stress. Across all strains, Chromosome IV emerged as a TE hotspot, likely reflecting shared genomic characteristics that tolerate or facilitate transposition in this region.

Increased outcrossing enhances recombination rates, creating genomic instability that can activate TE mobilization (Arkhipova & Meselson, 2000). Additionally, the introduction of genetic variation through outcrossing may temporarily disrupt TE silencing mechanisms, further contributing to higher TE activity in CB4856 (Hollister & Gaut, 2009). These results indicate that while higher outcrossing may promote the generation of genetic diversity, it can also trigger processes that increase susceptibility to mutagenic stress. This susceptibility may stem from strain-specific genomic architectures, the accumulation of deleterious mutations, or the difficulty of purging large-scale genomic changes under conditions of frequent outcrossing.

In conclusion, we found strain specific fitness and mutation responses which may reflect larger patterns of evolutionary response. Although the N2 “Bristol” strain, originally collected by Sydney Brenner (Brenner 1974) has been developed as a model system, its fitness, behavior and genomics differ substantially from other strains of *C. elegans*. N2 is a highly lab adapted strain with little genetic variation, few males and low outcrossing frequencies (Sterken et al., 2015). In contrast, CB4856 and AB1 are more recently collected “wild worms” with substantial genomic differences (Thompson et al., 2015) and high natural variation in male frequency (Anderson et al., 2010) and outcrossing (Teotonio et al., 2012). Additionally, our study reveals that SVs can act as genomic traps for SNPs thereby shielding these mutations from purging through recombination and outcrossing. This mechanism was most prominent in outcrossing-prone strains, suggesting that higher rates of recombination may not always facilitate genome cleanup, particularly for large or complex mutations. The natural genetic variation in *Caenorhabditis* remains a powerful resource for dissecting how genome architecture and mating systems interact to shape evolutionary outcomes.

## Methods

### Strains and mutagens

Three *C. elegans* strains with varying male frequencies were chosen: N2 (Bristol), CB4856 (Hawaiian), and AB1. ST2 served as a common competitor in relative fitness assays. All strains were obtained from the Caenorhabditis Genetics Center and maintained frozen until the experiment. Frozen strains were thawed on 75 mm × 13 mm NGM plates seeded with *E. coli* OP50. After one generation (^~^3.5 days at 201°C) for recovery, each strain was divided into nine replicate populations. Prior to the experiment, replicates were synchronized by bleaching. Over five generations, four replicates per strain were mutagenized with formaldehyde, four with EMS, and one remained untreated as a non-mutagenized control. All replicates for a given strain were handled in parallel on the same day using the same batch of plates to minimize intra-strain variability due to plate effects. Following the mutagenesis phase, all populations were frozen and stored at −801°C for downstream analyses.

### Mutagenesis

Four replicates per strain were treated with 1 mM ethyl methanesulfonate (EMS) in M9 solution, while the other four replicates were treated with 0.1% (w/v) formaldehyde solution in M9. These concentrations were chosen because they are non-lethal yet sufficient to induce detectable genomic mutations. During both the mutagenesis and recovery phases, worms were maintained on standard laboratory plates. However, during the mutagenesis phase, worms were suspended in mutagen solutions for 4 hours every fourth day before being transferred back to standard plates. In contrast, during the recovery phase, worms were not exposed to the mutagen and were allowed to grow normally on standard laboratory plates. We followed the worm mutagenesis protocol described by Brenner et al. (1974) with minor modifications. Every generation (^~^4 days), worms were washed from the plates with M9, collected in tubes, and resuspended in their respective mutagen solutions for 4 hours at 20 °C, during which they were gently rocked to ensure uniform exposure. After treatment, worms were transferred to fresh plates with NGM and *E. coli* OP50, ensuring maintenance of replicate identity and avoidance of mixing.

Population sizes were not altered at any point during the experiment. During both transfers, from the plates to the tubes and from the tubes back to the plates, all worms were transferred using 300 microliters of liquid. Specifically, M9 buffer was used for the transfer from plates to tubes, and the mutagen was used for the transfer from tubes back to plates. After the mutagen treatment, the liquid was carefully transferred onto the plate, causing parts of the *E. coli* colony to be dislodged. As a result, the worms would initially swim on the plate. We then left the plate open, allowing the liquid to be absorbed within 30-40 minutes, after which the worms resumed crawling on the plate.

### Male frequency assay

To evaluate the impact of five generations of mutational exposure on male frequency, a male frequency assay was conducted using both non-mutagenized (parent) worms and worms immediately after five generations of mutagen treatment. Worms from each replicate population were bleached for age synchronization. The lifetime progeny production of worms was monitored by placing a single L4 worm on a 75mm x 13mm Petri plate, transferring it to a fresh plate every 24 hours. After 96 hours, the worm was discarded, and the progeny produced over the four days were sexed and counted. Any worms that died during the transfer process were excluded from the analysis.

### Outcrossing frequency assay

As with the male frequency assay, outcrossing frequency was assessed using the same set of replicate plates. The lifetime progeny production of each worm was monitored by placing a single L4 worm on a 75mm x 13mm Petri plate, transferring both male and hermaphrodite to a fresh plate every 24 hours. After 96 hours, both the male and hermaphrodite were discarded, and the progeny produced over the four days were allowed to grow to adulthood.

Outcrossing in *C. elegans* is known to produce approximately 50% male progeny (Yin & Haag, 2019). Based on male frequency assays, we determined that spontaneous male production in the N2 and AB1 strains, both before and after mutagenesis, was relatively low. Thus, plates for these strains were visually scanned for males, and plates with male frequencies exceeding the expected spontaneous range (1%–5%) and approaching 50% were categorized as outcrossing plates. For the CB4856 strain, which naturally exhibits higher baseline male frequencies (15–17%), male frequency was calculated for each plate. A threshold of 25% males was set, and plates with male frequencies meeting or exceeding this value were marked as outcrossing plates.

### Relative fitness assay

After five generations of mutagenesis, worms were frozen at −80°C and later recovered for relative fitness assay at sixth generation- representing the fitness immediately after five generations of mutagenesis and again after three generations of recovery from mutations using the standard *C. elegans* maintenance protocol at 20 °C. During recovery, worms were maintained using the standard worm maintenance protocol. For the relative fitness assay, one early-stage L1 worm from each focal strain (N2, CB4856, AB1) was paired with one L1 worm from the non-mutagenized common competitor strain ST2 expressing green fluorescent protein (GFP). Both strains were co-cultured on NGM plates with *E. coli* OP50 for eight days (^~^2 generations) at 20 °C. Washed worms from replicate plates (11 per generation/mutant/strain combination) were collected in M9 on a slide, imaged with and without a GFP filter on a fluorescent microscope, and counted using a Python-based code specific for worm morphology (available on the Github). Plates with zero worms of either type were excluded from analysis, assuming that the worm did not survive to lay eggs. Images captured without the GFP filter counted all worms present on the plate- both the focal strain and the common competitor strain. Conversely, images captured with the GFP filter only revealed the green, fluorescent competitor worms, allowing for estimation of their abundance relative to the total worm population.

Fitness was compared across non-mutagenized, post-mutagenized, and recovered lineages using automated image analysis (Supplementary Material Table 2). To validate the accuracy of the image analysis pipeline, we randomly selected a subset of samples and manually counted individuals. This confirmed that the automated method produced reliable counts, with no observed false positives or false negatives. We found that three generations of recovery from mutagenesis were sufficient for each strain to recover pre-mutagenesis fitness levels for both the mutagens (Figure 1B).

### DNA extraction and Sequencing

To assess the genomic impact of mutagenesis, high molecular weight DNA was extracted from worms (after 3 generations of recovery) using the Cytiva kit (detailed procedure available in the Supplementary Materials). Before starting out the DNA extraction we washed a minimum of ^~^10,0000 worms with M9 buffer from the plate into a tube and kept the worms rocking overnight to remove associated microbiome. Pooled PacBio long-read sequencing was performed for parental worms of all three strains (N2, CB4856, AB1) and one population per strain per mutagen. Additionally, Illumina short-read sequencing was conducted for each replicate of each treatment (control, EMS, formaldehyde) for all strains. This strategy enabled comprehensive analysis of genomic variants induced by mutagenesis and their phenotypic consequences in subsequent generations.

For the comprehensive genomic analysis, we sequenced a total of 36 samples. Using PacBio long-read sequencing, with an average depth of coverage of 94.52x, we sequenced nine samples: the three parental strains (N2, CB4856, AB1) as controls, and six mutagenized replicates i.e., one replicate per strain treated with each mutagen. Additionally, we performed Illumina short-read sequencing on 27 treatment replicates, achieving an average depth of coverage of 72.11x. These 27 samples included the three parental strains, 12 EMS-treated replicates, and 12 formaldehyde-treated replicates, with four replicates per treatment for each strain.

The male frequency and outcrossing rate assays were conducted on worms derived from the same batch that underwent five generations of mutagenesis. In contrast, the worms used for the relative fitness assay and DNA extraction came from a different set, although they were mutagenized in the same manner as the first batch.

### Population simulations

To quantitatively examine the influence of outcrossing on SV purging and retention we implemented our theoretical model in population simulations using the software SLiM 4.3 (Haller & Messer, 2023). Our model described a genomic system where mutations occur as SVs, either insertions, deletions or inversions. Each individual in a population of size *K* carried *n* structural mutations. We constructed dioecious populations containing equal numbers of males and females and self-fertile populations with a proportion of individuals reproducing through outcrossing and a proportion reproducing through selfing. Each population started at a specified size *K* and evolved through overlapping generations with reproduction and fitness differences creating variable population sizes. Each individual had a diploid chromosome 10,000 nucleotides in length. Recombination occurred along the chromosomes with uniform probability at a specified rate *r*. After reproduction SV mutations occurred at a rate μ and were removed at a rate ν, for example by back mutation via gene conversion (Katju et al., 2008).

We did not explicitly model the physical size of mutations but assumed that mutations had discrete phenotypic effect sizes that cumulatively influenced fitness. The probability of a SV mutation occurring at any location was uniform across the chromosome and each SV mutation conferred a small deleterious phenotypic effect drawn from Γ(k,θ) (Supplementary Figure 1; Eyre-Walker & Keightley, 2007; Keightley & Eyre-Walker, 2007; Racimo & Schraiber, 2014). Individual SVs interacted to decrease fitness faster-than-linearly and the fitness landscape had a negative curvature or synergistic epistasis (Supplementary Figure 2). The fitness of an individual carrying *n* SVs was $w(n)=exp^{-\left(an+\left(bn^{2}\right)/2\right)}$ where *a* and *b* were parameters determining the curvature of the fitness landscape (Charlesworth 1990). Our simulated populations evolved for 100,000 generations with the parameters *k* = 1, *θ* = 2, *μ* = 0.01, *ν* = 0.001, *a* = 0.001 and *b* = 0.0006. We evolved populations under a range of evolutionary scenarios including recombination rates *r*=0.5-1x10^−5^, *K*=100 to 5,000 and levels of self-fertility ranging from 0 to 100%.

### Data analysis

#### Male frequency assay

Male frequency was calculated as the ratio of male progeny to the total progeny laid by each worm, and this proportion was used as the unit of analysis. We assessed the normality of the data using the Shapiro-Wilk test and by visualizing its distribution with a histogram. As expected, the distribution was right-skewed, with a high proportion of zero values, indicating zero-inflation. Given the bounded nature (0 to 1) of the data and the excess zero values, we used a zero-inflated Beta regression for analysis. Since this model requires proportion values strictly between 0 and 1, we applied an epsilon adjustment by adding a small constant to all zero values to make the data more appropriate for the model. We performed the analysis using the glmmTMB package in R (R version 4.1.0, released 2021-05-18)(Bolker, 2022), implementing the following model:

Male Frequency~Strain×Mutagen

In this model Strain (N2, AB1, CB4856) and Mutagen treatment (EMS, Formaldehyde and non-mutagenized parents were included as fixed factors and an interaction term was added to check if the effect of strain on male frequency varied by mutagen identity. This model accounts for both (1) the continuous variation in male frequency and (2) the excess zero values separately, making it a better fit than standard regression models. Further we did Tukey’s HSD comparison (Abdi & Williams, 2010) using emmeans package in R (Searle et al., 1980).

#### Relative fitness assay

To quantify the relative fitness of the *C. elegans* strains N2, CB4856, and AB1, each plate was washed with M9 buffer solution to prepare the worms for imaging. Two aliquots from the washed plate were then placed onto a microscope slide and covered with a coverslip. We performed imaging to count the GFP-expressing competitor strain and the non-GFP-expressing focal strain worms within these aliquots. The allelic frequency of the non-fluorescent allele was calculated as the square root of proportion of non-GFP-expressing worms to the total worm population. For statistical analysis, two-way ANOVAs were conducted separately for each mutagen to assess the effects of strain and treatment duration on relative fitness. Data normality was examined using the Shapiro-Wilk test and the residual distribution showed slight variation from normality (Shapiro-Wilk test: W = 0.9868, *p* < 0.01), indicating variation from normality. However, visual inspection of the residuals with histogram suggested an approximately normal distribution. Since ANOVA is robust to minor deviations, we proceeded with ANOVA followed by pairwise comparisons using Tukey’s HSD test. The following linear model was applied:

anova(lm(RF~Strain*Generation,data=a)).

Following ANOVA, we performed pairwise comparisons using Tukey’s Honest Significant Difference (HSD) test to assess differences in relative fitness across strains and generations. We conducted separate Tukey HSD tests within each strain to compare relative fitness before mutagenesis, after five generations of mutagenesis, and after three generations of recovery.

#### Bioinformatic analysis

The bioinformatic analysis for identifying *de novo* mutations following PacBio sequencing involved a comprehensive workflow to process and analyse long-read data, distinguishing mutations that arose in the worms after mutagenesis from those existing in parental strains. Initially, raw PacBio reads were aligned and indexed to reference genomes using minimap2 (Li, 2018) and bcftools (Danecek et al., 2021); for the N2 and CB4856 strains, NCBI reference genomes were used, while the AB1 strain employed the recently annotated genome of its descendant strain, SX3368 (Riccio C, 2022), to account for genomic divergences within the lineage. Subsequent variant calling was performed using Sniffles (Smolka et al., 2024) for structural variants (SVs), such as insertions, deletions, and inversions, capitalizing on the accuracy offered by long-read data.

For the specific identification of de novo mutations induced by mutagenesis, a comparative analysis using bcftools contrasted the variant profiles of generation 8 worms (post three generations of recovery) with their control parents, filtering out pre-existing genetic variations. All parental and mutagenized samples were processed using identical library preparation, sequencing, and bioinformatics pipelines to ensure comparability in variant detection. Further quality control and data filtering ensured only high-quality SNPs and SVs (quality score ≥ 20, depth ≥ 10) were retained. We then used bedtools to provide insights into their potential impacts on exonic regions and regulatory elements.

Additionally, similar bioinformatic steps were conducted with Illumina sequencing data. Alignment and indexing were again performed on reference genomes, followed by SNP and SV calling using Freebayes (Ypsilanti et al.,2021)and Manta (Chen et al., 2016), respectively. We initially called variants in pooled-population mode (FreeBayes ‘pooled’) to account for minor alleles in the combined worm samples. However, when intersecting these SNP calls with structural variants, we observed inflated counts due to multi-allelic sites being represented multiple times. To avoid this over-counting artifact, we generated a separate VCF using non-pooled calling parameters and used these calls for all subsequent intersection and annotation analyses. Enhanced SV detection was achieved with VGtoolkit (Hickey et al., 2020) through graph-based variant calling, which was then integrated with SVs identified by Sniffles 2.0 using Survivor 1.0.3 (Jeffares et al., 2017), creating a comprehensive view of the genomic alterations induced by the mutagens.

Long-read sequencing was performed on the three non-mutagenized parent strains and one replicate per strain for each mutagen treatment. Short-read sequencing covered all replicates across all strains. For structural variation analysis, we merged variants identified by both long-read and short-read callers using Survivor 1.0.3. For SNP analysis, we relied solely on variants identified by short-read callers.

#### Annotation of Transposable Elements and Intersection with Structural Variants

Transposon annotation is facilitated in complete and contiguous sequences, thus annotation of TEs was done in the N2, AB, and CB reference genomes rather than directly from the raw sequence data. We used the TransposonUltimate reasonaTE (Riehl et al., 2022) pipeline for TE annotation in each of the reference genomes. Briefly, TEUltimate reasonaTE wraps 13 previously published TE annotation tools, which include: RepeatMasker v4.1.1 , RepeatModeler v2.0.1, SINE-Scan , SINE-Finder, LTRPred , LTRHarvest, TIRvish , MUSTv2 , MITE-Tracker, MITEFinderII, HelitronScanner, TransposonPSI for transposon discovery. While some functions are optional, all subprograms were run with default parameters in this study. The resulting annotations are filtered, merged, and clustered using CD-HIT v4.8.1 (Fu et al., 2012) and BLASTN v2.10.1 (Camacho et al., 2009). The final annotations from FinalAnnotations_Transposons.gff3 in the final Results folder were used for downstream analysis for each of the three focal strains.

Evidence of transposon movement was assumed from structural variants which overlapped with transposon annotations. The TEUltimatedeTEct pipeline (Riehl et al., 2022) was used for intersection of transposon and structural variant annotations. De novo VCF structural variant files, as generated above, were intersected with transposon annotations and reported as an event if overlapping by at least 10% and lengths were similar by at least 50%. Finally, any annotation smaller than 50 base pairs, or larger than 1% of the genome was discarded. Plots were generated from the transpositionEvents.gff3 file in RStudio with R v4.3.2 (R Core Team, 2023).

## Supplementary Material

Supplement 1

## Data Availability

Scripts, bioinformatic workflows and analyses are available at github.com/RohitKapila. Python code used for image analysis is available at github.com/FierstLab/WormImage. Fitness data are available as Supplemental Materials associated with the manuscript. Sequence reads are available at the NCBI SRA under BioProject PRJNA1201181.

## References

[R1] Abdi H., & Williams L. J. (2010). Principal component analysis. In Wiley Interdisciplinary Reviews: Computational Statistics (Vol. 2, Issue 4, pp. 433–459). 10.1002/wics.101

[R2] Anderson J. L., Morran L. T., & Phillips P. C. (2010). Outcrossing and the maintenance of males within C. elegans populations. Journal of Heredity, 101(SUPPL. 1). 10.1093/jhered/esq003 PMC2859890 20212008

[R3] Arkhipova I., & Meselson M. (2000). Transposable elements in sexual and ancient asexual taxa. Harvard School of Public Health. www.pnas.org 10.1073/pnas.97.26.14473 PMC18943 11121049

[R4] Artieri C. G., Haerty W., Gupta B. P., & Singh R. S. (2008). Sexual selection and maintenance of sex: Evidence from comparisons of rates of genomic accumulation of mutations and divergence of sex-related genes in sexual and hermaphroditic species of Caenorhabditis. Molecular Biology and Evolution, 25(5), 972–979. 10.1093/molbev/msn046 18281268

[R5] Bolker B. (2022). Getting started with the glmmTMB package.

[R6] Brenner S. (1974). The genetics of *Caenorhabditis elegans*. Genetics 77: 71–94. 4366476 10.1093/genetics/77.1.71 PMC1213120

[R7] Byers D. L., & Waller D. M. (1999). Do Plant Populations Purge Their Genetic Load? Effects of Population Size and Mating History on Inbreeding Depression. In Source: Annual Review of Ecology and Systematics (Vol. 30). https://about.jstor.org/terms

[R8] Camacho C., Coulouris G., Avagyan V., Ma N., Papadopoulos J., Bealer K., & Madden T. L. (2009). BLAST+: Architecture and applications. BMC Bioinformatics, 10. 10.1186/1471-2105-10-421 PMC2803857 20003500

[R9] Charlesworth B. (1990a). Mutation-selection balance and the evolutionary advantage of sex and recombination. Genetical Research, 55(3), 199–221. 10.1017/S0016672300025532 2394378

[R10] Charlesworth B. (1990b). OPTIMIZATION MODELS, QUANTITATIVE GENETICS, AND MUTATION. Evolution, 44(3), 520–538. 10.1111/j.1558-5646.1990.tb05936.x 28567983

[R11] Charlesworth B., & Charlesworth D. (1983). The population dynamics of transposable elements. Genetical Research, 42(1), 1–27. 10.1017/S0016672300021455

[R12] Chen X., Schulz-Trieglaff O., Shaw R., Barnes B., Schlesinger F., Källberg M., Cox A. J., Kruglyak S., & Saunders C. T. (2016). Manta: Rapid detection of structural variants and indels for germline and cancer sequencing applications. Bioinformatics, 32(8), 1220–1222. 10.1093/bioinformatics/btv710 26647377

[R13] Ciccia A., & Elledge S. J. (2010). The DNA Damage Response: Making It Safe to Play with Knives. In Molecular Cell (Vol. 40, Issue 2, pp. 179–204). 10.1016/j.molcel.2010.09.019 20965415 PMC2988877

[R14] Collins R. L., Brand H., Karczewski K. J., Zhao X., Alföldi J., Francioli L. C., Khera A. V., Lowther C., Gauthier L. D., Wang H., Watts N. A., Solomonson M., O’Donnell-Luria A., Baumann A., Munshi R., Walker M., Whelan C. W., Huang Y., Brookings T., … Talkowski M. E. (2020). A structural variation reference for medical and population genetics. Nature, 581(7809), 444–451. 10.1038/s41586-020-2287-8 32461652 PMC7334194

[R15] Danecek P., Bonfield J. K., Liddle J., Marshall J., Ohan V., Pollard M. O., Whitwham A., Keane T., McCarthy S. A., & Davies R. M. (2021). Twelve years of SAMtools and BCFtools. GigaScience, 10(2). 10.1093/gigascience/giab008 PMC7931819 33590861

[R16] Dolgin E. S., & Charlesworth B. (2006). The fate of transposable elements in asexual populations. Genetics, 174(2), 817–827. 10.1534/genetics.106.060434 16888330 PMC1602064

[R17] Estes S., Phillips P. C., Denver D. R., Thomas W. K., & Lynch M. (2004). Mutation Accumulation in Populations of Varying Size: The Distribution of Mutational Effects for Fitness Correlates in Caenorhabditis elegans. 10.1534/genetics.166.3.1269 PMC1470770 15082546

[R18] Eyre-Walker A., & Keightley P. D. (2007). The distribution of fitness effects of new mutations. In Nature Reviews Genetics (Vol. 8, Issue 8, pp. 610–618). 10.1038/nrg2146 17637733

[R19] Feuk L., Carson A. R., & Scherer S. W. (2006). Structural variation in the human genome. In Nature Reviews Genetics (Vol. 7, Issue 2, pp. 85–97). 10.1038/nrg1767 16418744

[R20] Fu L., Niu B., Zhu Z., Wu S., & Li W. (2012). CD-HIT: Accelerated for clustering the next-generation sequencing data. Bioinformatics, 28(23), 3150–3152. 10.1093/bioinformatics/bts565 23060610 PMC3516142

[R21] Gaertner B. E., & Phillips P. C. (2010). Caenorhabditis elegans as a platform for molecular quantitative genetics and the systems biology of natural variation. Genetics Research, 92(5–6), 331–348. 10.1017/S0016672310000601 21429266

[R22] García-Dorado A. (2012). Understanding and predicting the fitness decline of shrunk populations: Inbreeding, purging, mutation, and standard selection. Genetics, 190(4), 1461–1476. 10.1534/genetics.111.135541 22298709 PMC3316656

[R23] Glémin S. (2003). How are deleterious mutations purged? Drift versus nonrandom mating. Evolution, 57(12), 2678–2687. 10.1111/j.0014-3820.2003.tb01512.x 14761049

[R24] Haller B. C., & Messer P. W. (2023a). SLiM 4: Multispecies Eco-Evolutionary Modeling. American Naturalist, 201(5), E127–E139. 10.1086/723601 PMC10793872 37130229

[R25] Hartfield M. (2016). Evolutionary genetic consequences of facultative sex and outcrossing. Journal of Evolutionary Biology, 29(1), 5–22. 10.1111/jeb.12770 26431643

[R26] Hickey G., Heller D., Monlong J., Sibbesen J. A., Sirén J., Eizenga J., Dawson E. T., Garrison E., Novak A. M., & Paten B. (2020). Genotyping structural variants in pangenome graphs using the vg toolkit. Genome Biology, 21(1). 10.1186/s13059-020-1941-7 PMC7017486 32051000

[R27] Ho S. S., Urban A. E., & Mills R. E. (2020). Structural variation in the sequencing era. In Nature Reviews Genetics (Vol. 21, Issue 3, pp. 171–189). Nature Research. 10.1038/s41576-019-0180-9 PMC7402362 31729472

[R28] Hoffmann A. A., & Rieseberg L. H. (2008). Revisiting the impact of inversions in evolution: From population genetic markers to drivers of adaptive shifts and speciation? In Annual Review of Ecology, Evolution, and Systematics (Vol. 39, pp. 21–42). 10.1146/annurev.ecolsys.39.110707.173532 PMC2858385 20419035

[R29] Hollister J. D., & Gaut B. S. (2009). Epigenetic silencing of transposable elements: A trade-off between reduced transposition and deleterious effects on neighboring gene expression. Genome Research, 19(8), 1419–1428. 10.1101/gr.091678.109 19478138 PMC2720190

[R30] Jeffares D. C., Jolly C., Hoti M., Speed D., Shaw L., Rallis C., Balloux F., Dessimoz C., Bähler J., & Sedlazeck F. J. (2017). Transient structural variations have strong effects on quantitative traits and reproductive isolation in fission yeast. Nature Communications, 8. 10.1038/ncomms14061 PMC5286201 28117401

[R31] Katju V., LaBeau E. M., Lipinski K. J., & Bergthorsson U. (2008). Sex change by gene conversion in a Caenorhabditis elegans fog-2 mutant. Genetics, 180(1), 669–672. 10.1534/genetics.108.090035 18757925 PMC2535716

[R32] Keightley P. D., & Eyre-Walker A. (2007). Joint inference of the distribution of fitness effects of deleterious mutations and population demography based on nucleotide polymorphism frequencies. Genetics, 177(4), 2251–2261. 10.1534/genetics.107.080663 18073430 PMC2219502

[R33] Kirkpatrick M., & Barton N. (2006). Chromosome inversions, local adaptation and speciation. Genetics, 173(1), 419–434. 10.1534/genetics.105.047985 16204214 PMC1461441

[R34] Kondrashov A. S. (1982). Selection against harmful mutations in large sexual and asexual populations. Genetical Research, 40(3), 325–332. 10.1017/S0016672300019194 7160619

[R35] Konrad A., Thompson O., Waterston R. H., Moerman D. G., Keightley P. D., Bergthorsson U., & Katju V. (2017). Mitochondrial mutation rate, spectrum and heteroplasmy in Caenorhabditis elegans spontaneous mutation accumulation lines of differing population size. Molecular Biology and Evolution, 34(6), 1319–1334. 10.1093/molbev/msx051 28087770 PMC5850408

[R36] Kutscher L. M., & Shaham S. (2014). Forward and reverse mutagenesis in C. elegans. In WormBook : the online review of C. elegans biology (pp. 1–26). 10.1895/wormbook.1.167.1 PMC4078664 24449699

[R37] Li H. (2018). Minimap2: Pairwise alignment for nucleotide sequences. Bioinformatics, 34(18), 3094–3100. 10.1093/bioinformatics/bty191 29750242 PMC6137996

[R38] Limpose K. L., Corbett A. H., & Doetsch P. W. (2017). BERing the burden of damage: Pathway crosstalk and posttranslational modification of base excision repair proteins regulate DNA damage management. In DNA Repair (Vol. 56, pp. 51–64). Elsevier B.V. 10.1016/j.dnarep.2017.06.007 28629773 PMC5576989

[R39] Lynch M., Blanchard J., Houle D., Kibota T., Schultz S., Vassilieva L., & Willis J. (1999). Perspective: Spontaneous deleterious mutation. Evolution, 53(3), 645–663. 10.1111/j.1558-5646.1999.tb05361.x 28565627

[R40] Lynch M., Burger R., Butcher D., Gabriel W., Charlesworth B., Hansen T., Houle D., Kondrashov A., Lande R., & Willis J. (1993). The Mutational Meltdown in Asexual Populations. We are very grateful to. In Journal of Heredity (Vol. 84). 10.1093/oxfordjournals.jhered.a111354 8409355

[R41] MacPherson B., Scott R., & Gras R. (2021). Sex and recombination purge the genome of deleterious alleles: An Individual Based Modeling Approach. Ecological Complexity, 45. 10.1016/j.ecocom.2021.100910

[R42] Mahmoud M., Gobet N., Cruz-Dávalos D. I., Mounier N., Dessimoz C., & Sedlazeck F. J. (2019). Structural variant calling: The long and the short of it. In Genome Biology (Vol. 20, Issue 1). BioMed Central Ltd. 10.1186/s13059-019-1828-7 PMC6868818 31747936

[R43] Negi P., Rai A. N., & Suprasanna P. (2016). Moving through the stressed genome: Emerging regulatory roles for transposons in plant stress response. In Frontiers in Plant Science (Vol. 7, Issue OCTOBER2016). Frontiers Media S.A. 10.3389/fpls.2016.01448 PMC5056178 27777577

[R44] Ospina R., & Ferrari S. L. P. (2012). On Bias Correction in a Class of Inflated Beta Regression Models. International Journal of Statistics and Probability, 1(2). 10.5539/ijsp.v1n2p269

[R45] Otto S. P. (2008). Unravelling the evolutionary advantage of sex: A commentary on ‘Mutation-selection balance and the evolutionary advantage of sex and recombination’ by Brian Charlesworth. In Genetics Research (Vol. 89, Issues 5–6, pp. 447–449). 10.1017/S001667230800966X 18976534

[R46] Racimo F., & Schraiber J. G. (2014). Approximation to the Distribution of Fitness Effects across Functional Categories in Human Segregating Polymorphisms. PLoS Genetics, 10(11). 10.1371/journal.pgen.1004697 PMC4222666 25375159

[R47] Riccio C. (2022). Riccio C., 2022. Duplication is a prominent mechanism of recent gene birth in Caenorhabditis elegans (Doctoral dissertation).

[R48] Riddle DL B. T. M. B. ,. (1997). Mechanism of Tc1 Transposition. In Cold Spring Harbor Laboratory Press; : Vol. Section VI. Cold Spring Harbor Laboratory Press.

[R49] Riehl K., Riccio C., Miska E. A., & Hemberg M. (2022a). TransposonUltimate: software for transposon classification, annotation and detection. Nucleic Acids Research, 50(11), E64. 10.1093/nar/gkac136 35234904 PMC9226531

[R50] Robert F., & Pelletier J. (2018). Exploring the Impact of Single-Nucleotide Polymorphisms on Translation. In Frontiers in Genetics (Vol. 9). Frontiers Media S.A. 10.3389/fgene.2018.00507 PMC6218417 30425729

[R51] Searle S. R., Speed F. M., & Milliken G. A. (1980). Population marginal means in the linear model: An alternative to least squares means. American Statistician, 34(4), 216–221. 10.1080/00031305.1980.10483031

[R52] Sianta S. A., Peischl S., Moeller D. A., & Brandvain Y. (2023). The efficacy of selection may increase or decrease with selfing depending upon the recombination environment. Evolution, 77(2), 394–408. 10.1093/evolut/qpac013 36622723

[R53] Smolka M., Paulin L. F., Grochowski C. M., Horner D. W., Mahmoud M., Behera S., Kalef-Ezra E., Gandhi M., Hong K., Pehlivan D., Scholz S. W., Carvalho C. M. B., Proukakis C., & Sedlazeck F. J. (2024). Detection of mosaic and population-level structural variants with Sniffles2. Nature Biotechnology. 10.1038/s41587-023-02024-y PMC11217151 38168980

[R54] Sterken M. G., Snoek L. B., Kammenga J. E., & Andersen E. C. (2015). The laboratory domestication of Caenorhabditis elegans. In Trends in Genetics (Vol. 31, Issue 5, pp. 224–231). Elsevier Ltd. 10.1016/j.tig.2015.02.009 25804345 PMC4417040

[R55] Szövényi P., Devos N., Weston D. J., Yang X., Hock Z., Shaw J. A., Shimizu K. K., McDaniel S. F., & Wagner A. (2014). Efficient purging of deleterious mutations in plants with haploid selfing. Genome Biology and Evolution, 6(5), 1238–1252. 10.1093/gbe/evu099 24879432 PMC4041004

[R56] Teotonio H., Carvalho S., Manoel D., Roque M., & Chelo I. M. (2012). Evolution of outcrossing in experimental populations of caenorhabditis elegans. PLoS ONE, 7(4). 10.1371/journal.pone.0035811 PMC3335146 22540006

[R57] Thompson O. A., Snoek L. B., Nijveen H., Sterken M. G., Volkers R. J. M., Brenchley R., van’t Hof A., Bevers R. P. J., Cossins A. R., Yanai I., Hajnal A., Schmid T., Perkins J. D., Spencer D., Kruglyak L., Andersen E. C., Moerman D. G., Hillier L. D. W., Kammenga J. E., & Waterston R. H. (2015). Remarkably divergent regions punctuate the genome assembly of the Caenorhabditis elegans hawaiian strain CB4856. Genetics, 200(3), 975–989. 10.1534/genetics.115.175950 25995208 PMC4512556

[R58] Whitlock M. C., & Agrawal A. F. (2009a). Purging the genome with sexual selection: Reducing mutation load through selection on males. In Evolution (Vol. 63, Issue 3, pp. 569–582). 10.1111/j.1558-5646.2008.00558.x 19154364

[R59] Wright S. I., & Schoen D. J. (1999). Transposon dynamics and the breeding system. In Genetica (Vol. 107). 10952207

[R60] Ypsilanti A. R., Pattabiraman K., Catta-Preta R., Golonzhka O., Lindtner S., Tang K., Jones I. R., Abnousi A., Juric I., Hu M., Shen Y., Dickel D. E., Visel A., Pennacchio L. A., Hawrylycz M., Thompson C. L., Zeng H., Barozzi I., Nord A. S., & Rubenstein J. L. (2021). Transcriptional network orchestrating regional patterning of cortical progenitors. 10.1073/pnas.2024795118/-/DCSupplemental PMC8713794 34921112

[R61] Yin D., & Haag E. S. (2019). Evolution of sex ratio through gene loss. Proceedings of the National Academy of Sciences of the United States of America, 116(26), 12919–12924. 10.1073/pnas.1903925116 31189601 PMC6601293

