# Supplementary material for: Outcrossing Complicates Mutation Purging by Trapping Single Nucleotide Polymorphisms in Structural Variant Mutations
Source: bioRxiv. 2025 Aug 13:2025.04.16.649179. Preprint. [Version 2] doi: 10.1101/2025.04.16.649179 (PMC12363826; doi:10.1101/2025.04.16.649179)
Supplement: Supplement 1 [file media-1.docx]

**Supplementary Materials**

1. ***Protocols***

**DNA Extraction Protocol – Sera-Xtracta HMW DNA Kit**

Preparation

- Wash the *C. elegans* plates thoroughly with M9 buffer to collect worms.
- Transfer the worm suspension to a tube and rock gently overnight at room temperature to allow removal of surface-associated microbiota.
- Centrifuge the suspension to pellet the worms.
- Carefully discard the supernatant without disturbing the pellet.
- Add fresh M9 buffer to the pellet and repeat the wash to further remove residual bacteria.
- Centrifuge again and discard the supernatant.
- Resuspend the final worm pellet in 200 µL M9 buffer in a microcentrifuge tube.
- Add 0.6 µL of 10% SDS solution to lyse the worms.
- Subject the samples to five freeze–thaw cycles:
- Freeze at –80 °C, then thaw at 25 °C, repeating this process five times to enhance lysis.

.

# Sample Lysis

- 1. Add 20 µL Proteinase K to the bottom of a 2 mL microcentrifuge tube.
- 2. Add 200 µL of worm sample
- 3. Add 200 µL Lysis buffer and vortex for 15 seconds.
- 4. Incubate at 25°C for 30 minutes (no agitation).
- 5. Vortex briefly for 5 seconds post incubation.
- 6. Briefly centrifuge to collect the lysate at the bottom of the tube.

# DNA Binding

- 1. Add 15 µL magnetic bead suspension (vortex before use).
- 2. Add 230 µL Binding buffer and vortex for 5 seconds.
- 3. Incubate at 25°C for 3 minutes at 1400 rpm.
- 4. Briefly centrifuge and place the tube on a magnetic rack for 1 minute.
- 5. Carefully aspirate and discard the supernatant.

# Washing (4 total washes)

- 1. Remove the tube from the magnetic rack.
- 2. Add 700 µL Wash 1 buffer. Vortex or shake to disperse beads.
- 3. Incubate at 25°C for 1 minute at 1400 rpm.
- 4. Centrifuge briefly and place on magnetic rack. Remove supernatant.
- 5. Repeat steps 1–4 with Wash 1 buffer once more.
- 6. Repeat steps 1–4 twice with Wash 2 buffer.

# Drying (Optional but recommended)

- 1. Briefly centrifuge to collect droplets.
- 2. Place on magnetic rack and aspirate any residual wash buffer.
- 3. Air dry the bead pellet for 5 minutes.

# Elution

- 1. Remove from magnet and add 100 µL Elution buffer.
- 2. Vortex to mix and dislodge pellet.
- 3. Incubate at 25°C for 3 minutes at 1400 rpm.
- 4. Place on magnetic rack for 1 minute.
- 5. Transfer the eluted DNA (supernatant) to a clean tube.

# Storage

- Store purified DNA at 4°C for short-term use.
- For long-term storage, aliquot and freeze at -20°C or lower.
- Avoid repeated freeze-thaw cycles.

**Supplementary Table 1. Pairwise contrasts of male frequency between *C. elegans* strains (AB1, CB4856, N2) and treatments (non-mutagenized parental controls, EMS, and formaldehyde).**

| **Contrast** | **estimate** | **SE** | **z.ratio** | **p.value** |
| --- | --- | --- | --- | --- |
| AB1 EMS - CB4856 EMS | -0.305 | 0.409 | -0.746 | 0.9981 |
| AB1 EMS - N2 EMS | 0.1547 | 0.395 | 0.392 | 1 |
| AB1 EMS - AB1 Formaldehyde | -0.021 | 0.401 | -0.052 | 1 |
| AB1 EMS - CB4856 Formaldehyde | -0.4269 | 0.427 | -1 | 0.986 |
| AB1 EMS - N2 Formaldehyde | 0.2724 | 0.394 | 0.691 | 0.9989 |
| AB1 EMS - AB1 Parent | 0.0301 | 0.36 | 0.083 | 1 |
| AB1 EMS - CB4856 Parent | -1.4614 | 0.358 | -4.085 | 0.0015 |
| AB1 EMS - N2 Parent | 0.1396 | 0.348 | 0.401 | 1 |
| CB4856 EMS - N2 EMS | 0.4597 | 0.387 | 1.188 | 0.9592 |
| CB4856 EMS - AB1 Formaldehyde | 0.284 | 0.394 | 0.722 | 0.9985 |
| CB4856 EMS - CB4856 Formaldehyde | -0.1219 | 0.42 | -0.291 | 1 |
| CB4856 EMS - N2 Formaldehyde | 0.5774 | 0.387 | 1.493 | 0.8591 |
| CB4856 EMS - AB1 Parent | 0.3351 | 0.352 | 0.952 | 0.9899 |
| CB4856 EMS - CB4856 Parent | -1.1564 | 0.348 | -3.319 | 0.0254 |
| CB4856 EMS - N2 Parent | 0.4446 | 0.339 | 1.31 | 0.9287 |
| N2 EMS - AB1 Formaldehyde | -0.1757 | 0.379 | -0.464 | 0.9999 |
| N2 EMS - CB4856 Formaldehyde | -0.5816 | 0.406 | -1.433 | 0.8852 |
| N2 EMS - N2 Formaldehyde | 0.1177 | 0.371 | 0.317 | 1 |
| N2 EMS - AB1 Parent | -0.1246 | 0.335 | -0.372 | 1 |
| N2 EMS - CB4856 Parent | -1.6161 | 0.333 | -4.859 | <.0001 |
| N2 EMS - N2 Parent | -0.0151 | 0.322 | -0.047 | 1 |
| AB1 Formaldehyde - CB4856 Formaldehyde | -0.4059 | 0.412 | -0.985 | 0.9873 |
| AB1 Formaldehyde - N2 Formaldehyde | 0.2934 | 0.378 | 0.776 | 0.9975 |
| AB1 Formaldehyde - AB1 Parent | 0.0511 | 0.343 | 0.149 | 1 |
| AB1 Formaldehyde - CB4856 Parent | -1.4404 | 0.34 | -4.236 | 0.0008 |
| AB1 Formaldehyde - N2 Parent | 0.1606 | 0.33 | 0.487 | 0.9999 |
| CB4856 Formaldehyde - N2 Formaldehyde | 0.6993 | 0.406 | 1.724 | 0.7317 |
| CB4856 Formaldehyde - AB1 Parent | 0.457 | 0.373 | 1.226 | 0.9509 |
| CB4856 Formaldehyde - CB4856 Parent | -1.0345 | 0.369 | -2.804 | 0.1141 |
| CB4856 Formaldehyde - N2 Parent | 0.5665 | 0.361 | 1.57 | 0.8212 |
| N2 Formaldehyde - AB1 Parent | -0.2423 | 0.335 | -0.724 | 0.9985 |
| N2 Formaldehyde - CB4856 Parent | -1.7338 | 0.333 | -5.213 | <.0001 |
| N2 Formaldehyde - N2 Parent | -0.1328 | 0.321 | -0.413 | 1 |
| AB1 Parent - CB4856 Parent | -1.4915 | 0.291 | -5.125 | <.0001 |
| AB1 Parent - N2 Parent | 0.1095 | 0.279 | 0.393 | 1 |
| CB4856 Parent - N2 Parent | 1.601 | 0.276 | 5.803 | <.0001 |

Supplementary Material Table 2: Pairwise comparisons of relative fitness across non-mutagenized, post-mutagenized, and recovered lineages for each strain and mutagen treatment.

| **Strain** | **Mutagen** | **Comparison** | **Difference (diff)** | **Lower Bound (lwr)** | **Upper Bound (upr)** | **P-Value (p adj)** |
| --- | --- | --- | --- | --- | --- | --- |
| N2 | Formaldehyde | Non-mutagenised - Mutagenised | -0.1207 | -0.2298 | -0.0116 | 0.02639 |
| N2 | Formaldehyde | Recovered - Mutagenised | -0.1166 | -0.2118 | -0.0213 | 0.01235 |
| N2 | Formaldehyde | Recovered - Non-mutagenised | 0.0041 | -0.1012 | 0.1095 | 0.99518 |
| N2 | EMS | Non-mutagenised - Mutagenised | -0.0554 | -0.1657 | 0.0549 | 0.45756 |
| N2 | EMS | Recovered - Mutagenised | -0.0541 | -0.1554 | 0.0472 | 0.41321 |
| N2 | EMS | Recovered - Non-mutagenised | 0.0012 | -0.1098 | 0.1123 | 0.99961 |
| CB4856 | Formaldehyde | Non-mutagenised - Mutagenised | 0.0283 | -0.0841 | 0.1406 | 0.82078 |
| CB4856 | Formaldehyde | Recovered - Mutagenised | 0.0282 | -0.0915 | 0.1479 | 0.84065 |
| CB4856 | Formaldehyde | Recovered - Non-mutagenised | -4.00E-05 | -0.1076 | 0.1075 | 0.99999 |
| CB4856 | EMS | Non-mutagenised - Mutagenised | 0.0081 | -0.1011 | 0.1173 | 0.98291 |
| CB4856 | EMS | Recovered - Mutagenised | -0.0736 | -0.1903 | 0.0432 | 0.29531 |
| CB4856 | EMS | Recovered - Non-mutagenised | -0.0817 | -0.1897 | 0.0264 | 0.17516 |
| AB1 | Formaldehyde | Non-mutagenised - Mutagenised | -0.0014 | -0.1279 | 0.1251 | 0.9996 |
| AB1 | Formaldehyde | Recovered - Mutagenised | 0.015 | -0.0961 | 0.1261 | 0.94408 |
| AB1 | Formaldehyde | Recovered - Non-mutagenised | 0.0164 | -0.0986 | 0.1314 | 0.93766 |
| AB1 | EMS | Non-mutagenised - Mutagenised | 0.0931 | -0.0369 | 0.223 | 0.20632 |
| AB1 | EMS | Recovered - Mutagenised | 0.1276 | 0.0155 | 0.2397 | 0.02183 |
| AB1 | EMS | Recovered - Non-mutagenised | 0.0345 | -0.0817 | 0.1507 | 0.75683 |

Supplementary Material Table 3: Nucleotides affected by structural variations for each strain

| **Strain** | **Mutagen** | **Insertion** | **Deletion** | **Duplication** | **Inversion** |
| --- | --- | --- | --- | --- | --- |
| N2 -1 | Formaldehyde | 471238 | 74044 | 44318 | 1289368 |
| N2 -2 | Formaldehyde | 471238 | 74044 | 44318 | 1289368 |
| N2 -3 | Formaldehyde | 471238 | 74044 | 44318 | 1289368 |
| N2 -4 | Formaldehyde | 471238 | 74044 | 44318 | 1289368 |
| N2 -1 | EMS | 482307 | 56936 | 36357 | 2642474 |
| N2 -2 | EMS | 482307 | 56936 | 36357 | 2642474 |
| N2 -3 | EMS | 482307 | 56936 | 36357 | 2642474 |
| N2 -4 | EMS | 482307 | 56936 | 36357 | 2642474 |
| CB4856-1 | Formaldehyde | 960440 | 118305 | 1169792 | 49702760 |
| CB4856-2 | Formaldehyde | 960182 | 118422 | 1169792 | 49702760 |
| CB4856-3 | Formaldehyde | 960237 | 118305 | 1169792 | 49702760 |
| CB4856-4 | Formaldehyde | 960409 | 118422 | 1169792 | 49702760 |
| CB4856-1 | EMS | 1011363 | 152738 | 21170220 | 32829363 |
| CB4856-2 | EMS | 1011427 | 152738 | 21170220 | 32829363 |
| CB4856-3 | EMS | 1011363 | 152738 | 21170220 | 32829363 |
| CB4856-4 | EMS | 1011363 | 152738 | 21170220 | 32829363 |
| AB-1 | Formaldehyde | 35468 | 46363 | 0 | 2539375 |
| AB-2 | Formaldehyde | 35468 | 46363 | 0 | 2539375 |
| AB-3 | Formaldehyde | 35468 | 46363 | 0 | 2539375 |
| AB-4 | Formaldehyde | 35468 | 46363 | 0 | 2539375 |
| AB-1 | EMS | 28269 | 22975 | 4640 | 4181603 |
| AB-2 | EMS | 28269 | 22975 | 4640 | 4181603 |
| AB-3 | EMS | 28269 | 22975 | 4640 | 4181603 |
| AB-4 | EMS | 28269 | 22975 | 4640 | 4181603 |
